# Workplace Sexual Harassment and the Risk of Chronic Disease in a Prospective Cohort Study

**DOI:** 10.3390/bs16020223

**Published:** 2026-02-03

**Authors:** Sally Freels, Tracy W. Lin, Timothy P. Johnson, Kathleen M. Rospenda

**Affiliations:** 1 Division of Epidemiology and Biostatistics, School of Public Health, University of Illinois at Chicago, Chicago, IL 60612, USA; 2 Department of Psychiatry, University of Illinois at Chicago, Chicago, IL 60612, USA; tracy.lin127@gmail.com (T.W.L.); timj@uic.edu (T.P.J.); rospenda@uic.edu (K.M.R.); 3 National Opinion Research Center, University of Chicago, Chicago, IL 60603, USA

**Keywords:** sexual harassment, chronic disease incidence, proportional hazards regression

## Abstract

In a sample of university employees, longitudinal data were examined to test a biopsychosocial model of whether exposure to workplace sexual harassment increases hazard for chronic disease, in the context of other known biological, psychological, and social risk factors for chronic disease. Proportional hazards multiple regression was used to predict incidence of first chronic disease across 23 years of follow-up based on experience of sexual harassment. Out of a sample of N = 525, 288 incident diagnoses were observed. Effects of harassment, drinking behavior, cigarette use, depressive symptoms, anxiety, and other work stressors were considered as either fixed at baseline or as time-dependent covariates in separate models, controlling for age and baseline occupational group, which were significantly associated with disease onset. Higher scores on reported workplace sexual harassment at baseline of the study were predictive of chronic disease incidence over the next 23 years (HR = 1.038 for each increase of one unit, *p* = 0.0133), adjusting for age and occupation. The effect was only partially attenuated when adjusting for depressive symptoms at baseline and alcohol intake throughout follow-up (HR = 1.031, *p* = 0.0475), the only other covariates tested that were consistently associated with chronic disease onset and included in final models. Considering the binary comparison of any versus no harassment at baseline revealed a stronger effect on chronic disease onset (HR = 1.437, *p* = 0.004), which again was attenuated after considering effects of baseline depressive symptoms and previous year alcohol use (HR = 1.357, *p* = 0.017). Experience of sexual harassment in the workplace was the only work stressor found to be significantly associated with an elevated risk of chronic disease onset across the study period.

## 1. Introduction

Workplace sexual harassment represents unwelcome sexual conduct that affects an individual’s job status or creates a hostile or offensive working environment (Equal Employment Opportunity Commission, 1980). Research evidence over the past two decades has supported an association between the experience of workplace sexual harassment (SH) and indicators of poor health (see Willness et al., 2007 for meta-analysis). From a strictly biomedical model, this would suggest that this association results from “disordered somatic (biochemical or neurophysiological) processes” (Engel, 1977, p. 130). By contrast, the biopsychosocial model recognizes the simultaneous contributions of biological, psychological, and social phenomena to health (Engel, 1977; Bolton, 2023). In this study, the biopsychosocial model is used to frame an examination of the relative impact of sexual harassment on the onset of chronic disease over a 23 year period, in the context of known biological, psychological, and social factors associated with health.

The biopsychosocial model was proposed by Engel (1977) as a reaction to the biomedical model, which viewed the body and mind as separate entities, with bodily processes driven by chemistry and physics alone, unrelated to psychosocial issues. Engel instead argued that physical and psychosocial issues cannot be viewed in isolation, and that doing so would both distort perspectives on the meaning of disease and interfere with patient care, citing the growing body of evidence that psychosocial conditions influence susceptibility to a wide variety of diseases in both animal and human studies (Engel, 1977).

Using the biopsychosocial model as a framework to examine the impact of SH on chronic disease onset, it is important to recognize that SH is conceptualized as both a psychological and a social phenomenon, involving cognitive processes whereby individuals differently perceive and respond to harassment, as well as social factors such as gender norms, workplace culture, and power differentials that structure why SH occurs and how it is defined (see Liang, 2024 for a review). A similar argument can be made for other job-based stressors, such as psychological workload and control over decisions, which derive their status as stressors based on both individual perceptions and workplace conditions. The present study contributes to occupational health research by recognizing that the effects of occupational stressors, such as SH, on health should be considered within the context of other biological, psychological, and social factors that can be associated with health and with the stressor itself. For the present study, SH represents the focal stressor, but the effects of psychological workload and decision latitude (i.e., job control) are also considered. Psychological workload and decision latitude are components of job strain, which has been linked to the hazard of developing chronic disease (Sørensen et al., 2021), particularly coronary heart disease in studies of longer than 10 years (Xu et al., 2015).

In the present study, the effects of the *biological* variables alcohol use and cigarette use are considered alongside the effects of SH given existing evidence of their association with SH and/or health outcomes. Alcohol research has consistently shown that SH is associated with increases in alcohol use and misuse (Richman et al., 2002; Rospenda et al., 2017; Rospenda et al., 2009), and with alcohol-related morbidity and mortality (Blindow et al., 2023). A causal association has been established between alcohol use and cancer risk (Boffetta & Hashibe, 2006; LoConte et al., 2018). Heavy drinking is additionally associated with risk for heart disease, stroke, and cirrhosis of the liver (see Rehm et al., 2017 for a review). In terms of cigarette use, there is some evidence that smoking may increase for adolescents on days when sexual harassment occurs (Livingston et al., 2022), although research with adults has found little association (e.g., Okechukwu et al., 2010; Seelig et al., 2017). There is a well-established link, however, between cigarette smoking and a variety of chronic diseases, including cardiovascular disease and cancer (Dai et al., 2022; Tasdighi et al., 2025).

In terms of *psychological* variables, depressive symptoms and anxiety are considered in the present study, given their documented associations with both SH and health outcomes. A large body of longitudinal research has accumulated documenting associations between SH and symptoms of depression and anxiety (see Chan et al., 2008; Diez-Canseco et al., 2022; Willness et al., 2007 for reviews). Rugulies and colleagues found evidence suggesting that SH precedes depression, showing that onset of workplace sexual harassment in the prior 12 months was associated with incidence of depressive disorder in a sample of almost 10,000 Danish workers (Rugulies et al., 2020). Depression has also been linked to the onset of chronic health conditions, primarily development or worsening of cardiac issues (Ferketich et al., 2000; Pratt et al., 1996) and diabetes (Eaton et al., 1996; Graham et al., 2020). Similarly, anxiety has been found to be modestly associated with cardiovascular disease onset (Wu et al., 2022), more strongly when both anxiety and depression are present (Nakada et al., 2025).

Finally, the present study considers *social* factors that are known to be associated with health, namely age, gender, race/ethnicity, and occupation. There is a strong consensus that aging is linked to the onset of disease, with increase in chronic inflammation over time representing a likely underlying factor (Baechle et al., 2023). Gender is included as a variable of interest because SH research typically takes place within a gendered framework, finding women at greater risk than men, although effects of SH do not consistently differ by gender (e.g., Blindow et al., 2024). Gender is also a correlate of disease, with men generally being more susceptible to heart disease (Crimmins et al., 2019) and substance use disorders (Vasilenko et al., 2017), and women generally exhibiting higher prevalence of autoimmune disease (Ngo et al., 2014), arthritis, and depression in most countries (Crimmins et al., 2019). There is a large body of research documenting health disparities associated with race/ethnicity (Cullen et al., 2022), with differences mostly ascribed to unequal social conditions and racism leading to poorer health (Paradies et al., 2015). Finally, occupation frames exposure to a variety of conditions that can impact health. SH is one such exposure, which is patterned by occupation and job–gender context, with national data from the US showing a higher prevalence for women in construction/extraction and production/transportation jobs, which tend to be male dominated (Rospenda et al., 2009).

The present study addresses an additional limitation of prior research, in that research shows that, when it occurs, SH tends to be a chronic issue (McGinley et al., 2011), with the consequent potential for longer term impacts on health. Yet studies of the association between SH and physical health in the U.S. tend to be cross sectional, focusing on self-reported acute health symptoms that are generally more time-limited, for example, headaches, gastrointestinal complaints, and problems sleeping (Magley et al., 1999; Wasti et al., 2000; Willness et al., 2007), rather than diagnosed chronic health conditions as outcomes of exposure to harassment. It is important to learn how SH may contribute to the development of chronic diseases, which develop over time, are enduring rather than reversible, and require long-term management or monitoring, with the four most common types being cardiovascular disease, cancer, chronic respiratory diseases such as asthma, and diabetes (World Health Organization, 2016). Research evidence is growing in support of a significant association between SH and chronic disease, namely, hypertension (Thurston et al., 2019), cardiovascular disease (Jakubowski et al., 2021; Prakash et al., 2024), and type 2 diabetes, particularly for those with frequent SH exposure (Prakash et al., 2024). In the first study to examine long-term health effects of exposure to SH among both women and men who were initially sampled from a U.S. Midwestern urban university, Abdulla and colleagues (Abdulla et al., 2023) tested the impact of chronic SH exposure on incidence of disease. “Chronic” SH was defined in prior research as membership in a latent class characterized by higher initial levels of SH at baseline, and a constantly higher level of SH over ten years of data collection, compared to those in a class of those with infrequent SH exposure (McGinley et al., 2011). Abdulla et al. (2023) found that exposure to a pattern of prior chronic SH between 1996 and 2006 was associated with increased odds of being diagnosed with arthritic or rheumatic conditions during the study period ending in 2020.

In this paper, analyses expand on data previously reported in Abdulla et al. (2023) by testing a data analytic approach that allows for SH exposure to vary over time. Additionally, a limitation of existing research is addressed by examining the impact of SH as measured by responses to a multi-item scale to capture a range of exposure to harassing events. This is important because prior research has often assumed that participants can accurately recognize and label their experiences as sexual harassment. Research has found that SH can negatively impact targets’ health even if they do not directly label their experiences as sexual harassment (Magley et al., 1999). Here, the association of SH with diagnosis of chronic disease was examined across a 23-year study period, accounting for other biological, psychological, and social factors known to be associated with chronic disease, with the goal of selecting a model that most accurately and parsimoniously explained the associations between tested variables and onset of chronic disease. It is hypothesized that exposure to SH will be associated with onset of chronic disease during the study period, independently of other covariates that display significant associations with chronic disease onset. The predictive value of SH as a time-varying variable versus a binary indicator at baseline is also considered.

## 2. Materials and Methods

*Recruitment and Study Population:* Study participants were originally recruited in 1996 from a sample of 4832 employees of an urban Midwestern university in the United States. The investigative team were researchers from the same university, none of whom were affiliated with university administration. The original purpose of this prospective cohort study was to examine workplace harassment as a risk factor for alcohol use and misuse, in the context of other psychosocial stressors and life experiences. The sample was stratified equally by both gender (50% women, 50% men) and occupational group (faculty, graduate student workers/trainees, clerical/administrative workers, service/maintenance workers). Occupational groups were selected to ensure a broad representation of different occupations, as most SH research at the time was being conducted on samples of students and faculty only. All participants had access to medical insurance and the Employee Assistance Program or student mental health services support during their employment at the university.

During each of 9 waves of data collection (see Table 1 for a description of the study timetable), respondents were contacted by mail and invited to complete and return self-administered paper questionnaires. During T9, respondents were additionally contacted by web (where possible) and invited to complete the questionnaire online. The surveys conducted during each survey wave closely followed the Dillman et al. (2014) Tailored Design Method, which included personalized outreach, multiple contact attempts and additional follow-up reminder prompts to non-respondents (including e-mail and telephone contacts), and carefully constructed survey questionnaires that followed best practices for instrument design.

At baseline (survey period from October 1996 to February 1997; T1), N = 2492 individuals responded to the survey (52% response rate). Study participants completed questionnaires asking about their experiences of workplace harassment, symptoms of depression, and alcohol use at baseline and then at eight additional time points through February of 2021 (T9). Surveys were administered at approximately 12 month intervals between 1996 and 2008, with the exceptions being gaps of three years between the 1998 and 2001 surveys, two years between the 2003 and 2005 surveys, and 12 years between the 2008 and 2020 surveys. Time point nine (June 2020–February 2021; T9) occurred on average 23 years following baseline. The T9 survey included questions about retrospective reports of chronic disease diagnosis, including age at first diagnosis. Those who responded at baseline were resurveyed throughout the entire study regardless of whether they remained employed by the university. A total of n = 920 responses were obtained at T9.

At all survey time points, potential respondents were provided with information about the study and potential risks and benefits of participation and asked to express consent by returning a completed questionnaire. Respondents were compensated at each wave, ranging from $20 at T1 to $50 at T9. The T1–T8 surveys were conducted by the University of Illinois at Chicago Survey Research Laboratory, and the T9 survey was conducted by the University of Chicago Survey Lab. The study was approved by the University of Illinois at Chicago Institutional Review Board (protocol 2019-0374); the UIC IRB approval was endorsed by the NORC at the University of Chicago IRB.

The analysis sample (n = 525) is derived from all subjects who retrospectively reported age at first diagnosis when surveyed at T9, who had not had any chronic disease diagnosis yet at one year after baseline, and who were not missing baseline values on the measure of SH. To maintain timing consistent with causality, risk of new chronic disease diagnosis in any given year is predicted from time-dependent measures reported one year earlier; therefore, the observed event times, or first diagnoses, start one year after baseline.

*Workplace Stressors (Social–Psychological Variables):* Workplace sexual harassment experiences (SEQ) in the 12 months prior to each survey were assessed using a version of the 19-item Sexual Experiences Questionnaire (Fitzgerald, 1996; Champaign, IL, USA) with wording modified to be applicable to both men and women. Respondents indicated the frequency of experience of gender harassment (e.g., told suggestive stories or offensive jokes), unwanted attention (e.g., unwanted attempts to stroke or fondle you), and sexual coercion (e.g., implied faster promotions or better treatment if you were sexually cooperative) on a 3-point scale: 0 = never, 1 = once, or 2 = more than once. Coefficient alpha reliability for the full scale was 0.80 or higher at each data collection point.

Other work stressors were measured with the five-item Psychological Demands (PSYCHWL) and nine-item Decision Latitude (DECLAT) scales of the Job Content Questionnaire (Karasek & Theorell, 1990; Lowell, MA, USA). Psychological Demands represent the extent to which one views themselves as having a heavy workload, conflicting demands, and insufficient task completion time. Decision Latitude assesses the extent to which individuals perceive themselves as having a say in how work is carried out, and the extent to which the job involves creativity and learning new things. Coefficient alpha for each scale was 0.72 or higher at each data collection point.

*“Biological” Variables:* Number of drinks consumed per day (DRINKS) was measured at each survey time point with the item, “When you drank any type of alcoholic beverage during the last 30 days, how many drinks did you usually have per day? (One drink would be equal to one 5-ounce glass of wine, one can/bottle of beer, or one shot of whiskey/hard liquor.)” Responses were reported on a scale from 0 = none/never drank in the past 30 days to 7 = more than 6 drinks (Cahalen et al., 1969; New Brunswick, NJ, USA).

Frequency of drinking (ALCDAYS) was measured at each survey point with the item, “During the last 30 days, on about how many days did you drink any type of alcoholic beverage?”(Cahalen et al., 1969; New Brunswick, NJ, USA).

Drinking to intoxication (INTOX) in the past 12 months was measured at each survey time point with the item, “About how often in the past 12 months did you drink enough to feel drunk, that is, when drinking noticeable affected your thinking, talking or behavior?” Response options ranged from 0 = never in those 12 months to 7 = 5 times a week or more (Wilsnack et al., 1991; Grand Forks, ND, USA).

Problem drinking/alcohol misuse was measured by the 10-item Brief Michigan Alcoholism Screening Test (BMAST), a brief screening measure for alcohol misuse derived from the full-length Michigan Alcoholism Screening Test (Pokorny et al., 1972; Houston, TX, USA). The instrument was scored continuously to capture a spectrum of alcohol misuse symptoms, rather than using cut-off scores to categorize the sample into levels of misuse.

Use of cigarettes (CIGS) was captured at each data collection point with the item, “In the past 12 months, have you used any traditional cigarettes or tobacco that you light and smoke?” (1 = yes, 0 = no).

*Psychological Variables*: Depressive symptoms (DEPR) in the past 7 days were measured at each survey time point with seven items from the Center for Epidemiologic Studies Depression scale (CESD) (e.g., I could not shake off the blues; I had trouble keeping my mind on what I was doing), which correlate highly with the full CESD (Mirowsky & Ross, 1990; Champaign, IL, USA). Items were scored on a scale from 1 = rarely or none of the time (under 1 day), to 4 = most or all of the time (5–7 days). Higher scores indicate greater symptoms of depression. Coefficient alpha reliability for the overall scale was 0.80 or higher at each data collection point.

Anxiety (ANX) in the past 7 days was measured at each time point with 9 items from the Profile of Mood States (McNair et al., 1981; Pearson Clinical Assessments, PO Box 599700, San Antonio, TX, 78259, USA). Each symptom (e.g., tense, panicky, nervous) is rated on a scale from 0 = not at all to 4 = extremely. Coefficient alpha reliability for the overall scale was 0.85 or higher at each data collection point.

*“Social” Variables:* Current age was included as a time-dependent variable calculated from age at baseline and current survey year. Occupational group at baseline (1 = clerical/administrative; 2 = faculty; 3 = graduate student workers; 4 = service/maintenance) was included as both a social variable and a design characteristic of the study. Race/ethnicity (White, Black, Hispanic, Asian) and gender (male, female) were included as fixed nominal variables representing factors that are associated with social position and are known correlates of health.

*Chronic Disease Incidence:* Age at first diagnosis of cardiovascular disease, asthma, cancer, diabetes, or arthritic disease is calculated from retrospective self-reports of chronic disease diagnosis history at the wave of follow-up. The number of years from baseline to first chronic disease diagnosis is calculated from age at baseline and age at first diagnosis reported for those with observed event times. Right-censored event times are number of years from baseline until last measurement wave for those reporting no chronic disease diagnoses. Diagnoses were combined into a single outcome for several reasons. First, incidence of individual disease was infrequent in some cases, so power limitations precluded testing of diseases in isolation. Second, use of a single outcome prevented the need to test a large number of models, which would require adjustment for multiple comparisons. Third, workplace stressors are thought to have systemic biological effects, namely, low-grade chronic inflammation, which contribute to the development of multiple chronic conditions (Nakata, 2012; Wright et al., 2020). As such, aggregation of diseases into a single outcome better captures the overall systemic effects of harmful exposures, which is of primary concern in the present study, as opposed to examining effects on specific diseases. Finally, in the context of occupational health, consideration of any chronic disease that may negatively affect job performance (e.g., absenteeism) is more meaningful to employers than consideration of specific conditions, which often overlap (e.g., those with hypertension often may also have other cardiovascular issues).

*Statistical Analysis:* All statistical analyses were performed in SAS version 9.4 (SAS, 100 SAS Campus Drive, Cary, NC, USA). In order to ensure that changes in repeated measures precede incidence, the hazard at each year after baseline was modeled as a function of independent variables during the previous year. This required elimination of those with any chronic disease already diagnosed up to and including baseline. The full dataset has 8 waves across the first 11 years after baseline (1996–1997), followed by a 12 year gap, and with another wave of data collection at year 23. First chronic disease diagnosis events were analyzed for years 1 through 23 after baseline, with all remaining observations right-censored after year 23.

Proportional hazards multiple regression was used to model incidence of first chronic disease diagnosis as a function of fixed and time-varying measures during the previous year. The model requires that each measure is assigned a value for each observed time of incidence in the dataset. Time-varying measures had various size gaps between measurements up to year 11, and a 12-year gap from the last measurement to the final wave at year 23. The assumption adopted here was that each measure remained constant until a new measurement was collected, or last value carried forward.

The effect of sexual harassment (SEQ) on chronic disease incidence was examined first using models with fixed independent variables only to facilitate estimation of cumulative incidence for descriptive purposes. The original sample was stratified across four occupation groups, and age is an obvious factor as well, so both age and occupation group were included in all models. Survival curves in SEQ subgroups were estimated and displayed, adjusting for age and occupation group at baseline.

Next, optimal thresholds for current age as time-dependent covariates calculated from age at baseline were used to more accurately adjust for current age across time. In the interest of parsimony, gender and race/ethnicity were considered and added to models with occupation at baseline, current age, and SEQ, but only if they had significant independent effects or if their adjustment resulted in substantial change to the estimated effect of SEQ.

Moving on to time-dependent covariates based on time-varying measures, the effect of SEQ as a fixed effect at baseline was compared to the effect of SEQ as a time-varying covariate, adjusting for all selected confounders at baseline. Additional time-varying measures considered in the analysis were depressive symptoms (DEPR); anxiety symptoms (ANX); various measures of alcohol use—average drinks per day past 30 days (DRINKS), times intoxicated past 12 months (INTOX), days drank past 30 days (ALCDAYS), and Brief MAST problem drinking scale past 12 months (BMAST); cigarette smoking past 12 months (CIGS); psychological workload (PSYCHWL); and decision latitude (DECLAT). Each measure added to the model required elimination of observations missing the measure at baseline; therefore, variable selection was performed adding each measure one at a time to the model with SEQ and selected social variables (age and occupation), together with re-estimation of the SEQ effect with the smaller sample size and without the new measure for comparison. Each measure was considered as fixed at baseline and as time-varying with last value carried forward. Other variables whose effects were not statistically significant at α = 0.05 were excluded to preserve power. A final model is reported with selected independent variables.

To examine the assumption of proportional hazards across the entire follow-up period, which is essentially an assumption of no interaction with time, we tested a model with conditional effects of SEQ for different time intervals.

## 3. Results

### 3.1. Baseline Characteristics

Table 2 reports characteristics of the analysis sample at baseline. A total of 525 subjects reported retrospective history of chronic disease 23 years after baseline, reported no chronic disease diagnosis yet at baseline, and were not missing SEQ (the focal predictor) at baseline. A total of 1967 were excluded. Baseline mean age of the analysis sample was 38.3 years (SD = 9.6). Over half of the sample were women, and a majority were white. Most of those in the analysis sample were either from the faculty or graduate student worker groups at baseline, with the smallest number being from the service/maintenance group. A majority (67.6%) were married at T9 (5.0% widowed, 12.6% divorced, 1.7% separated, and 12.2% never married). Regarding employment status, 74.1% worked for pay at some point in the 12 months prior to the T9 survey, and 82.3% were no longer employed at the university (17.3% remained employed at the university). Information about differences between the analysis sample, the T9 sample excluded from analysis, and overall excluded sample are reported in Supplementary Tables S4 and S5. Briefly, the analysis sample differed from those with T9 survey data who were excluded in the following ways: the analysis sample tended to be younger at baseline (*p* < 0.001), were more likely to be white or Asian (*p* < 0.005), and were less likely to be from the clerical/administrative occupational group and more likely to be from the graduate student worker group at baseline (*p* < 0.001). The analysis sample did not differ from the T9 sample excluded in terms of SH experience (y/n) or SEQ score.

### 3.2. Chronic Disease Incidence

Of the 525 included cases, there were 288 cases with first diagnoses reported ranging from 1 year to 23 years after baseline. Median time to first diagnosis was 21.0 years after baseline (95% C.I. [20, 23]). By T9, the average age of the analysis sample was 48.7 (SD = 9.4, see Table 3). The four most common chronic diseases as noted by the WHO—cardiovascular disease, cancer, chronic respiratory diseases such as asthma, and diabetes—were also four of the most common new diagnoses represented in the sample. Additionally, a high rate of new onset of arthritic disease was found. The rates of new onset of these diseases in the sample differed in some respects from the prevalence of these diseases in the US population of those aged 45 or older. The most common first chronic disease diagnosis in the sample was cardiovascular disease, which included hypertension (48%, versus 61%+ prevalence nationally; Joynt Maddox et al., 2024), followed by arthritic disease (27%, versus 26%+ prevalence nationally; Fallon et al., 2023), cancer (16%, versus 5.2%+ prevalence nationally; National Center for Health Statistics, 2023), diabetes (9%, versus 14.5% prevalence nationally; Centers for Disease Control and Prevention, 2024), and asthma (6%, versus 8.2% prevalence nationally; Centers for Disease Control and Prevention, 2023). Percentages sum to more than 100% across the 288 diagnoses due to some multiple first diagnoses during the same year.

### 3.3. Initial Models Testing SEQ at Baseline Only

**Figure 1:**
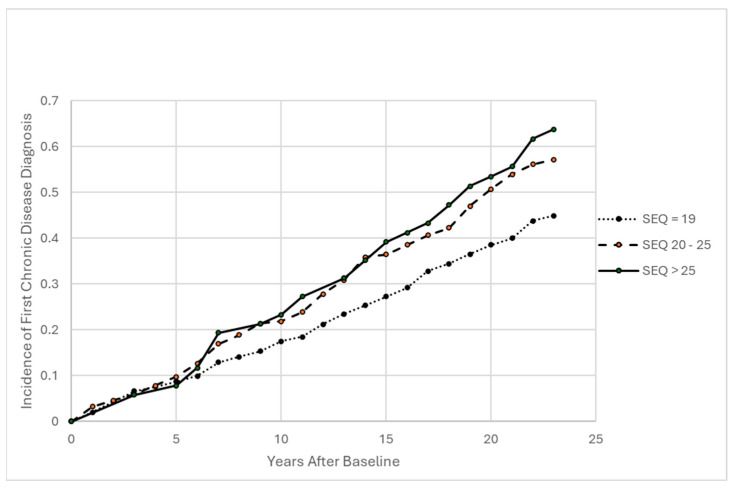
First Chronic Disease Incidence By SEQ at Baseline, Adjusted for Age and Occupation Group at Baseline (N = 525; 288 Incident Diagnoses). Note: Estimated cumulative incidence for age = 38 (sample mean) and occupation group = faculty modeled as proportional hazards covariates, stratified by SEQ group (SEQ = 19, n = 284, 142 events; SEQ 20–25, n = 192, 114 events; SEQ > 25, n = 49, 32 events).

Before exploring time-varying measures, an initial model for incidence of first chronic disease as a function of SEQ at baseline was fit, exhibiting a statistically significant trend for the linear effect of SEQ (HR = 1.035, *p* = 0.0163), adjusting for age at baseline and occupation group. This suggests that each increase of one unit in SEQ is associated with a 3.5% increase in annual incidence of chronic disease. Modeling SEQ instead as a binary indicator for any versus no harassment reported (SEQ > 19 versus SEQ = 19) is also significant, suggesting a nearly 50% increase in hazard with any harassment (HR = 1.468, *p* = 0.0018). In order to provide a picture of observed survival trends, three categories were defined for SEQ, and a proportional hazards model was fit stratified on SEQ categories and adjusted for age and occupation group at baseline. The estimated underlying baseline hazard from this model was used to portray observed cumulative incidence (calculated as “1 minus survival”) in the three groups (Figure 1).

### 3.4. Model Building, Testing Possible Covariates

Model selection was performed in parallel for models with a single linear effect reflecting amount of harassment, and models with a single binary indicator for any harassment. When examining models allowing time-dependent covariates, the parametrization of current age most associated with chronic disease incidence was found to be a set of two indicator variables, one for current age ≥ 40 and one for current age ≥ 50. These two variables for current age, as well as occupation group at baseline, were included in all models.

The next step was consideration of time-varying measures. Table 3 describes potentially health-relevant measures available in the data, including SEQ, within the set of subjects still at risk for first chronic disease diagnosis, at each time point where new measures were taken. Given the amount of data missing across time and a lack of clear trends, the assumption of measurements remaining constant until they are measured again, last value carried forward (McNight et al., 2007) was implemented for modeling. Available alternative methods substituting estimates based on individual or group trends in the sample are not easily incorporated into the proportional hazards framework.

Details of all models fit to perform variable selection are included as Supplementary Tables. Supplementary Table S1 is a summary of first steps to select whether SEQ fixed vs. time-dependent was most informative, as well as selection of demographic variables to include for adjustment. The estimated effect of each increase of one unit of the SEQ scale fixed at baseline is HR = 1.037 (*p* = 0.0091), and HR = 1.033 (*p* = 0.0249) for SEQ time-varying. The effect fixed at baseline is slightly stronger in magnitude, and also has a smaller standard error, resulting in a lower p-value when tested against zero. The effect of a binary indicator for SEQ > 19 as fixed at baseline is HR = 1.492 (*p* = 0.0011) and time-varying is HR = 1.189 (*p* = 0.1588). Here, the effect fixed at baseline is far stronger than time-varying, which is no longer statistically significant. SEQ, both continuous for a single trend and as a binary indicator of any harassment, was defined in all further models as fixed at baseline.

Additional social variables, gender and race/ethnicity, were considered. Indicator variables for female (versus male), as well as indicators for each sizeable race/ethnicity category (White, Black, Hispanic, and Asian) added one at a time to the model, did not exhibit independent statistically significant effects, and did not substantially alter the estimated effect of SEQ and were thus excluded from the final models.

Next, each of the time-varying measures was considered one at a time, first as fixed at baseline, then as time-varying with last value carried forward (Supplementary Table S2). For each measure, a slightly lower sample size was used, and the effect of SEQ before adjustment was fit on the smaller sample for comparison. The same process was completed separately for continuous SEQ and for binary SEQ > 19. For both measures, only two variables considered were found to have their own significant independent effects - depressive symptoms (DEPR) at baseline, and a time-varying covariate for average drinks per day (DRINKS). Table 4 shows details of the final selected models. Effects of continuous SEQ and binary SEQ > 19 remain significant and slightly less in magnitude adjusted for depressive symptoms at baseline, suggesting that depressive symptoms account for a small portion of the total effect. The independent significant effect of DRINKS as a time-varying measure accounts for a small portion of the effect of depressive symptoms but does not appear to impact the effects of SEQ at baseline. Additionally, older age was associated with increased hazard of disease, and membership in the faculty or graduate student occupational group at baseline was associated with reduced hazard of disease compared to the service/maintenance group. Full models that include the effects of age and occupation can be found in Supplementary Table S3.

**Figure 2:**
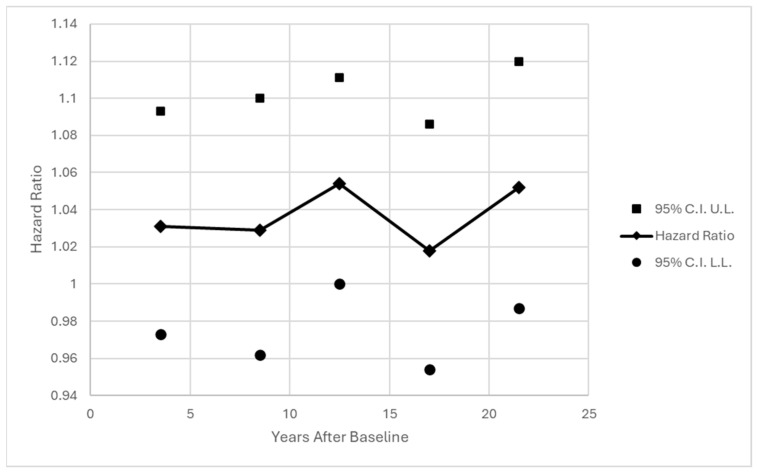
Effect of Continuous SEQ at Baseline on Hazard of First Chronic Disease Diagnosis within Time Intervals; Adjusted for Current age and Occupation at Baseline. Note: Intervals are 1–6 years (67 diagnoses); 7–10 years (50 diagnoses); 11–14 years (59 diagnoses); 15–19 years (63 diagnoses); and 20–23 years (49 diagnoses).

**Figure 3:**
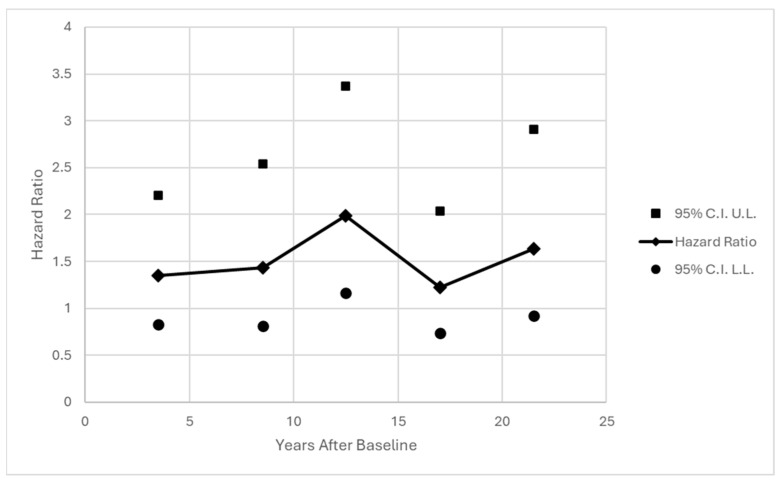
Effect of Binary SEQ > 19 at Baseline on Hazard of First Chronic Disease Diagnosis Within Time Intervals; Adjusted for Current Age and Occupation at Baseline. Note: Intervals are 1–6 years (67 diagnoses); 7–10 years (50 diagnoses); 11–14 years (59 diagnoses); 15–19 years (63 diagnoses); and 20–23 years (49 diagnoses).

One concern is the assumption of proportional hazards, or a consistent hazard ratio across time for the effect of SEQ at baseline, especially given the long follow-up period. Standard tests for interaction between time and SEQ did not reveal any evidence of monotonic trend across time (*p* = 0.5247 and *p* = 0.4148 for continuous SEQ, *p* = 0.6130 and *p* = 0.4655 for binary SEQ > 19, interactions with years and with log(years) respectively). A model was fit allowing for conditional effects of SEQ within each of five time intervals. Results are shown in Figure 2 and Figure 3. The effect of SEQ was relatively stable across time, and certainly not decreasing with distance from baseline.

## 4. Discussion

These analyses build on prior work demonstrating the long-term effects of exposure to chronic sexual harassment on onset of disease (Abdulla et al., 2023). The present study is unique in occupational health research on sexual harassment because of its consideration of a variety of possible co-contributors to chronic disease onset, including variables that impact biological processes (i.e., alcohol and cigarette use), psychological factors (i.e., depressive symptoms and anxiety), social factors (i.e., age, gender, race/ethnicity, occupation), and other social–psychological stressor variables (i.e., components of job strain) that are known correlates of disease. In order to conserve power, potential predictors were examined one at a time in parallel models as both fixed at baseline and time-dependent and those with significant effects independent of SH were selected. In addition to SH, age, occupation, depressive symptoms, and number of drinks consumed per day when drinking contributed significant predictive value in the final selected models. This supports a biopsychosocial perspective of disease, as indicators from biological, psychological, social, and social–psychological realms proved to be significantly associated with disease onset in this sample. Although testing of the specific mechanisms through which SH impacts chronic disease onset was beyond the scope of this paper, proposed pathways linking job stressors to health include dysregulation of immune or inflammatory responses (Wright et al., 2020); dysfunction of the hypothalamic–pituitary–adrenal (HPA) axis that can negatively impact cardiovascular, metabolic, and cognitive function (Ganster & Rosen, 2013); or behavioral pathways, including substance misuse or other unhealthy lifestyle choices (Siegrist & Rödel, 2006). Unfortunately, biomedical indicators of inflammation or other physical indicators of stress exposure (e.g., cortisol) that would allow for tests of physiological pathways were not collected in this study. Future large-scale longitudinal research collecting data on SH, health-related behaviors, mental health, and biomedical information over time will be needed to test potential mechanisms of disease progression, which will be challenging due to the need for sufficient power to examine multiple specific disease pathways.

In final models, alcohol consumption and depression were explored as both fixed covariates at baseline and as time-dependent covariates allowed to change across the follow-up period. Notably, the effect of alcohol consumption in the previous year remains significant in the final model, which is consistent with a growing body of literature documenting the relationship between alcohol use/misuse and both morbidity (e.g., Rehm et al., 2017) and mortality (e.g., Rehm & Shield, 2013). Significant independent effects for SH, depressive symptoms, and alcohol consumption suggest that models testing the mediating effects of depression and alcohol use on SH–chronic disease relationships might prove fruitful in helping to define disease progression pathways. While illicit drug use was not tested as a predictor in the current study, future research on larger samples should capture illicit drug use, which is known to be harmful to health.

The final models revealed several interesting findings regarding which variables best predicted chronic disease onset, and whether predictive value was stronger when predictors were treated as baseline indicators only or were considered as time-varying. When allowed to vary over time, SH and depressive symptoms proved to be weaker predictors than if considered at baseline only, despite the fact that both variables definitively vary over time. Although SH and depressive symptoms may be either acute or experienced over a longer period of time, in this sample it was the fact that they occurred at all at baseline that mattered for health.

Interestingly, only the measure of number of drinks consumed when drinking, but not measures of heavy or problem drinking, proved to be significantly associated with chronic disease onset, despite the fact that this measure may more likely be impacted by exposure to other, shorter-term stressors that were not measured in this study. Future research should assess a wider variety of stressors, including harassment that occurs outside of the workplace, discrimination, and general life stressors. Additionally, more frequent measurement points would allow for more complex modeling of the interplay between predictors of chronic disease over time.

While the lack of a significant effect for gender was somewhat surprising, this may be an artifact of the broad outcome measure of any chronic disease, such that possible gender differences in specific diseases were masked. Failure to detect significant effects for minority group membership may be a consequence of the smaller sample sizes and number of diagnoses available for each. It is also possible that the measure used in the present study fails to capture the full experience of sexual harassment for racial/ethnic minority group members. Prior intersectional research has demonstrated that women of color in particular may experience “racialized sexual harassment,” or harassment that combines aspects of both racism and sexism (Buchanan & Ormerod, 2002). Additionally, workers of color are more likely to experience other stressors and forms of discrimination (e.g., racial discrimination, institutionalized racism) (Williams, 2018) in the workplace, which were not captured in the present study. Future research is needed to examine the relative contribution of multiple distinct psychosocial hazards in the workplace, including sexual harassment, racial discrimination, workplace bullying, and other harmful exposures to worker health over time.

*Strengths:* Multiple repeated waves of data allowed for testing of models requiring exposure measurements to be completed at least one year prior to disease incidence. Resulting significant associations are more likely to be causal in nature. There were a wide variety of occupations represented in the sample, which included men, unlike much of the research on sexual harassment, and the sample was racially and ethnically diverse. Additionally, a variety of biological, psychological, social, and social–psychological predictors of health were tested for inclusion in final models, in line with the biopsychosocial model of health. Depressive symptoms and alcohol use, known correlates of both SH and health, were accounted for in final models, allowing for a more nuanced assessment of the independent impact of SH on development of chronic health conditions.

*Limitations:* The results, however, should be interpreted in the context of several limitations. The sample was originally drawn from a single urban Midwestern university in the United States, potentially limiting generalizability, and the analytic sample differed in some ways from those who were excluded from analyses, suggesting that the results should be interpreted cautiously as they may not be representative of the general working population. Additionally, recruitment of participants at baseline was conducted by a survey organization that was affiliated with the university, and study investigators were associated with the university. Respondents may have underreported SH, alcohol use, and depressive symptoms to the extent that they were uncomfortable disclosing such information to study investigators at the same institution or may have declined to participate in the study entirely. To help improve sense of anonymity, surveys were identified by unique ID number only. Another limitation is that, for those who changed jobs, information was not collected about the benefits and resources available to those individuals at their new jobs. Those who moved to jobs without health benefits, or with reduced benefits, may have been more likely to suffer adverse health outcomes. Also, the analytic sample size was relatively small, and subject to possible selection bias due to disproportionate dropout of those who experienced SH or developed/succumbed to chronic disease during the course of the study. The size of the analytic sample also limited power to detect significant associations with onset of disease, particularly among non-White racial/ethnic subgroups, and resulted in insufficient power to assess disease-specific associations. The inclusion of known risk factors for disease, although an improvement upon most existing studies, was still fairly limited. Future research would benefit from larger, representative samples, inclusion of additional known risk factors for chronic disease (e.g., BMI, physical activity, diet, use of medications, genetic risk factors), and more non-response follow-up to attempt to determine reasons for survey non-response.

Also, data were collected from self-report surveys and are subject to self-report bias, and there was a large gap in data collection between the original study (T1–T8) and follow-up at T9, leading to a loss of information about study variables during this intervening period. Additionally, diagnosis information was collected only at T9, and as such is at risk of recall bias, particularly for less recent diagnoses. Future research should investigate the long-term impacts of exposure to sexual harassment on health using more frequent data collection points and should attempt to corroborate reported diagnosis of chronic health conditions with data from medical records.

It should be noted that two significant events occurred during the study period that may have impacted chronic disease development, namely, the Great Recession from 2007–2009, and the COVID-19 pandemic, beginning in early 2020. Future research should attempt to account or control for events such as this in analyses, where possible. The present study also did not assess harassment occurring outside of the workplace, which is a limitation. Future research would benefit from more detailed exploration of exposure to sexual harassment by race/ethnicity, gender, and occupation, whether long-term health outcomes differ by these demographic characteristics, and how the course of exposure to harassment, both in and outside of the workplace, develops for different groups of individuals.

## 5. Conclusions

Despite study limitations, these results are important for several reasons. This is the first study to demonstrate that experience of sexual harassment in the workplace is associated with increased risk for subsequent onset of chronic disease in the context of other biological, psychological, and social predictors of disease, and this effect does not diminish over time. Age, occupation, depressive symptoms, and alcohol consumption are also independently associated with chronic disease incidence in the final models, suggesting the importance of broadening the scope of health-related covariates included in research studies so that the relative importance of various correlates is clearer. This is also important in the context of health promotion and disease prevention. Medical practitioners should include assessment of social–psychological health risks associated with one’s job, such as sexual harassment, when assessing cumulative risk for disease, along with depression and alcohol use, which are more typically already screened for in primary care settings. Also, organizations concerned with controlling healthcare costs and/or the productivity of a healthy workforce would be well advised to ensure that any reported sexual harassment is immediately addressed. Finally, the findings of this study suggest the importance of strengthening anti-sexual harassment public health campaigns to raise awareness and reduce the impact of this preventable workplace exposure on the health of the working population.

## Figures and Tables

**Table 1:** Basic survey information and timetable for each wave of data collection.

Survey Wave	Years Since Baseline	Dates	Eligible Sample	Completed Questionnaires	Response Rate (%)	Respondent Compensation
T1	0	10/1996–2/1997	4790	2492	52.0	$20
T2	1	11/1997–4/1998	2491	2038	81.8	$20
T3	5	5/2001–1/2002	2486	1730	69.6	$30
T4	6	9/2002–1/2003	2472	1654	66.9	$30
T5	7	11/2003–3/2004	2458	1453	59.1	$30
T6	8	9/2005–2/2006	2434	1517	62.3	$30
T7	9	9/2006–4/2007	2411	1461	60.6	$30
T8	10	10/2007–2/2008	2393	1444	60.3	$25
T9	23	6/2020–2/2021	2035	920	45.2	$50

**Table 2:** Characteristics of analysis sample (N = 525) at baseline.

Variable		n	%		
Age	≤30	130	26.6		
	31–40	167	34.2		
	41–50	128	26.3		
	>50	63	12.9		
		mean	SD	min	max
Age	years	38.3	9.6	22	68
		n	%		
Gender	Female	301	57.3		
	Male	224	42.7		
Race	White	340	64.7		
	Black	87	16.6		
	Hispanic	25	4.8		
	Asian	64	12.2		
	Other	9	1.7		
Occupation Group at Baseline	Clerical/Administrative	86	16.4		
	Faculty	205	39.0		
	Graduate Student	192	36.6		
	Service/Maintenance	42	8.0		
		n	%		
Any Sexual Harassment (SEQ)	=19 (none)	284	54.1		
	>19 (any)	241	45.9		
		mean	SD	min	max
Sexual Harassment Scale (SEQ)	19 to 57	21.3	3.9	19	45

**Table 3:** Time-varying measures.

Datapoints After BL =	0	1	3	4	5	6	7	8	9	10	11
Predicting Incidence in Year(s) =	1	2–3	4	5	6	7	8	9	10	11	12–23
Total at Risk for First Chronic Dx	525	511	486	479	470	458	435	427	417	408	399
Age (years)											
Age Mean	38.3	39.5	41.2	42.1	43.0	43.9	44.8	45.8	46.7	47.7	48.7
Age SD	9.6	9.6	9.4	9.4	9.3	9.3	9.4	9.4	9.4	9.4	9.4
Sexual Harassment (SEQ): Scale 19–57											
SEQ N	525	428	368	367	370	354	305	344	345	313	
new measure (X)	X	X			X	X		X	X	X	
retrospective (*)			*	*			*				
SEQ Mean	21.3	21.1	21.0	20.7	20.9	20.6	20.4	20.7	21.1	21.0	
SEQ SD	3.9	4.1	3.7	3.6	3.7	3.4	2.7	3.4	3.8	3.4	
% > 19	45.9	40.4	32.1	33.0	34.3	33.3	33.1	37.5	45.2	45.7	
Depressive Symptoms (DEPR): Scale 0–21											
DEPR N	503	444			383	388	332		354	359	339
DEPR Mean	3.23	3.92			3.53	3.60	3.06		3.19	3.20	3.09
DEPR SD	3.60	4.17			3.82	3.76	3.43		3.46	3.53	3.28
Drinks per Day Past 30 Days (DRINKS): Categories 0–7											
ANX: Scale 0–36											
ANX N	513	437			362	377	322		336	343	329
ANX Mean	7.9	7.5			6.8	7.0	6.2		6.7	6.3	6.5
ANX SD	6.2	6.0			5.4	5.7	5.1		5.3	5.1	5.1
DRINKS N	508	449			333	331	302		338	336	315
DRINKS Mean	1.40	1.32			1.32	1.24	1.28		1.28	1.29	1.37
DRINKS SD	1.33	1.26			1.07	1.10	1.05		1.03	1.06	1.13
Times Intoxicated (INTOX): Categories 0–7											
INTOX N	522	453			384	386	307		361	361	341
INTOX Mean	0.61	0.57			0.73	0.67	0.52		0.56	0.55	0.57
INTOX SD	1.03	0.94			1.19	1.10	0.88		0.99	1.05	1.05
Days Drank Past 30 Days (ALCDAYS): 0–30											
ALCDAYS N	517	449			381	382	332		352	347	328
ALCDAYS Mean	5.47	5.90			5.72	5.89	7.35		7.55	7.35	7.96
ALCDAYS SD	7.09	7.55			7.71	7.76	8.41		8.77	8.71	9.09
Alcohol Misuse (BMAST): Scale 0–17											
BMAST N	524	454			319	314	252		361	300	287
BMAST Mean	0.58	0.53			0.43	0.37	0.55		0.47	0.47	0.41
BMAST SD	1.52	1.46			1.19	1.42	1.49		1.30	1.37	1.17
Cigarette Smoking (CIGS): 0 = no, 1 = yes											
CIGS N	514	429			378	335	294		338	330	292
CIGS Mean	0.23	0.19			0.15	0.19	0.17		0.17	0.14	0.15
CIGS SD	0.42	0.39			0.36	0.39	0.37		0.37	0.35	0.36
Psychological Workload (PSYCHWL): Scale 0–25											
PSYCHWL N	491	416			330	317	276		341	314	
PSYCHWL Mean	10.2	9.6			9.5	9.9	9.5		9.8	9.9	
PSYCHWL SD	4.4	3.9			4.3	4.1	4.1		4.3	4.2	
Decision Latitude (DECLAT): Scale 12–60											
DECLAT N	511	421			343	322	282		351	315	
DECLAT Mean	38.4	38.5			39.1	38.9	38.6		39.0	39.2	
DECLAT SD	6.2	5.8			5.8	5.5	6.2		6.1	6.0	

**Table 4:** Proportional hazards multiple regression models for first chronic disease diagnosis (asthma, cancer, diabetes, cardiovascular or arthritic disease) (N = 488; 269 incident events, 219 right-censored; 23 years follow-up).

**Models with Single Trend Continuous SEQ**					
	**Beta**	**SE**	**Chi-Square**	***p*-Value**	**HR**
Model 1:					
SEQ at Baseline	0.03681	0.01487	6.1287	0.0133	1.038
Model 2:					
SEQ at Baseline	0.03029	0.01520	3.9693	0.0463	1.031
DEPR at Baseline	0.04766	0.01684	8.0082	0.0047	1.049
Model 3:					
SEQ at Baseline	0.03021	0.01524	3.9289	0.0475	1.031
DEPR at Baseline	0.04064	0.01714	5.6243	0.0177	1.041
DRINKS previous year	0.11966	0.04821	6.1609	0.0131	1.127
**Models with Binary Indicator SEQ > 19**					
	**Beta**	**SE**	**Chi-Square**	***p*-Value**	**HR**
Model 1:					
SEQ > 19 at Baseline	0.36272	0.12636	8.2404	0.0041	1.437
Model 2:					
SEQ > 19 at Baseline	0.30819	0.12808	5.7897	0.0161	1.361
DEPR at Baseline	0.04607	0.01692	7.4143	0.0065	1.047
Model 3:					
SEQ > 19 at Baseline	0.30532	0.12808	5.6827	0.0171	1.357
DEPR at Baseline	0.03966	0.01713	5.3610	0.0206	1.040
DRINKS previous year	0.11872	0.04811	6.0890	0.0136	1.126

Notes: Models adjusted for current age (≥40, ≥50) and occupation at baseline. SEQ = Sexual Harassment Scale (19–57). DEPR = Selected items from Center for Epidemiologic Studies Depression Scale (0–21). DRINKS = Drinks per day past 30 days (0–7 categories); value as reported for the previous year or, if missing, last previous reported value.

## Data Availability

Data used for analysis in this paper are available from the first author upon request.

## Supplementary Materials

The following supporting information can be downloaded at: https://www.mdpi.com/article/10.3390/bs16020223/s1, Supplementary Table S1. Model Results Used for Variable Selection: Demographics Proportional Hazards Multiple Regression Models for First Chronic Disease Diagnosis (asthma, cancer, diabetes, cardiovascular or arthritic disease) All Models Adjusted for Current Age (≥40, ≥50) and Occupation at Baseline N = 525; 288 Incident Events. Supplementary Table S2. Model Results Used for Variable Selection: Time-Varying Measures Proportional Hazards Multiple Regression Models for First Chronic Disease Diagnosis (asthma, cancer, diabetes, cardiovascular or arthritic disease) All Models Adjusted for Current Age (≥40, ≥50) and Occupation at Baseline. Supplementary Table S3. Parameter Estimates for Selected Models Proportional Hazards Multiple Regression Models for First Chronic Disease Diagnosis (asthma, cancer, diabetes, cardiovascular or arthritic disease) (N = 488; 269 incident events, 219 right-censored; 23 years follow-up). Supplementary Table S4. Characteristics of Included and Excluded Samples at Baseline of the Study (1996–1997). Supplementary Table S5. Statistical Tests Comparing Subsamples at Baseline Chi-square test results for categorical variables, independent samples t-test results for continuous variables.
