# Supplementary material for: Workplace Sexual Harassment and the Risk of Chronic Disease in a Prospective Cohort Study
Source: Behav Sci (Basel). 2026 Feb 3;16(2):223. doi: 10.3390/bs16020223 (PMC12937653; doi:10.3390/bs16020223)
Supplement: Supplementary file 1 [file behavsci-16-00223-s001.zip › behavsci-3987996-supplementary.pdf]

Workplace Sexual Harassment and the Risk of Chronic Disease in a Prospective Cohort Study

Sally Freels, Tracy W. Lin, Timothy P. Johnson, and Kathleen M. Rospenda

Supplemental Tables

SUPPLEMENTAL TABLE A.
Model Results Used for Variable Selection: Demographics
Proportional Hazards Multiple Regression Models for First Chronic Disease Diagnosis
(asthma, cancer, diabetes, cardiovascular or arthritic disease)
All Models Adjusted for Current Age (>=40, >=50) and Occupation at Baseline
N=525; 288 Incident Events

	HR	p-value
CHECK SEQ Baseline VS. TIME-DEPENDENT		
M1: Continuous SEQ at Baseline	1.037	.0091
M2: Continuous SEQ time dependent	1.033	.0249
M3: Indicator for SEQ>19 at Baseline	1.492	.0011
M4: Indicator for SEQ>19 time dependent	1.189	.1588
CHECK FEMALE		
M1: Continuous SEQ at Baseline	1.038	.0090
Female	0.884	.3173
M2: Indicator for SEQ>19 at Baseline	1.491	.0011
Female	0.886	.3260
CHECK RACE		
M1: Continuous SEQ at Baseline	1.037	.0094
White	1.043	.7695
M2: Indicator for SEQ>19 at Baseline	1.491	.0012
White	1.003	.9855
M3: Continuous SEQ at Baseline	1.036	.0116
Black	1.265	.1997
M4: Indicator for SEQ>19 at Baseline	1.503	.0009
Black	1.326	.1219
M5: Continuous SEQ at Baseline	1.037	.0092

Hispanic	0.836	.5280
M6: Indicator for SEQ>19 at Baseline	1.492	.0011
Hispanic	0.830	.5127
M7: Continuous SEQ at Baseline	1.037	.0102
Asian	0.907	.6580
M8: Indicator for SEQ>19 at Baseline	1.487	.0012
Asian	0.912	.6764

Note: SEQ = Sexual Harassment Scale (19-57).

SUPPLEMENTAL TABLE B.
Model Results Used for Variable Selection: Time-Varying Measures
Proportional Hazards Multiple Regression Models for First Chronic Disease Diagnosis
(asthma, cancer, diabetes, cardiovascular or arthritic disease)
All Models Adjusted for Current Age (>=40, >=50) and Occupation at Baseline

	Total N	Events	HR	p-value
SELECT NOT MISSING SEQ	525	288		
M1: Continuous SEQ at Baseline			1.037	.0091
M2: Indicator for SEQ>19 at Baseline			1.492	.0011
SELECT NOT MISSING SEQ, DEPR	503	278		
M1: Continuous SEQ at Baseline			1.040	.0078
M2: Continuous SEQ at Baseline			1.033	.0318
DEPR at Baseline			1.051	**.0023**
M3: Continuous SEQ at Baseline			1.035	.0189
DEPR time dependent			1.032	.0523
M4: Indicator for SEQ>19 at Baseline			1.474	.0019
M5: Indicator for SEQ>19 at Baseline			1.393	.0086
DEPR at Baseline			1.050	**.0034**
M6: Indicator for SEQ>19 at Baseline			1.444	.0033
DEPR time dependent			1.033	.0429
SELECT NOT MISSING SEQ, DRINKS	508	278		
M1: Continuous SEQ at Baseline			1.035	.0155
M2: Continuous SEQ at Baseline			1.035	.0153
DRINKS at Baseline			1.079	.0855
M3: Continuous SEQ at Baseline			1.033	.0219
DRINKS time dependent			1.149	**.0035**
M4: Indicator for SEQ>19 at Baseline			1.458	.0024
M5: Indicator for SEQ>19 at Baseline			1.446	.0030
DRINKS at Baseline			1.073	.1129

M6: Indicator for SEQ>19 at Baseline			1.433	.0038
DRINKS time dependent			1.146	**.0043**
SELECT NOT MISSING SEQ, ALCDAYS	517	284		
M1: Continuous SEQ at Baseline			1.036	.0116
M2: Continuous SEQ at Baseline			1.035	.0146
ALCDAYS at Baseline			1.008	.3807
M3: Continuous SEQ at Baseline			1.035	.0143
ALCDAYS time dependent			1.007	.3195
M4: Indicator for SEQ>19 at Baseline			1.499	.0010
M5: Indicator for SEQ>19 at Baseline			1.494	.0011
ALCDAYS at Baseline			1.009	.3265
M6: Indicator for SEQ>19 at Baseline			1.485	.0014
ALCDAYS time dependent			1.006	.3759
SELECT NOT MISSING SEQ, INTOX	522	287		
M1: Continuous SEQ at Baseline			1.037	.0105
M2: Continuous SEQ at Baseline			1.036	.0118
INTOX at Baseline			1.032	.6012
M3: Continuous SEQ at Baseline			1.036	.0124
INTOX time dependent			1.019	.7593
M4: Indicator for SEQ>19 at Baseline			1.488	.0012
M5: Indicator for SEQ>19 at Baseline			1.484	.0013
INTOX at Baseline			1.032	.5952
M6: Indicator for SEQ>19 at Baseline			1.483	.0014
INTOX time dependent			1.016	.7984
SELECT NOT MISSING SEQ, BMAST	524	288		
M1: Continuous SEQ at Baseline			1.037	.0097
M2: Continuous SEQ at Baseline			1.036	.0137
BMAST at Baseline			1.035	.3708
M3: Continuous SEQ at Baseline			1.035	.0172

BMAST time dependent			1.045	.2481
M4: Indicator for SEQ>19 at Baseline			1.486	.0012
M5: Indicator for SEQ>19 at Baseline			1.481	.0013
BMAST at Baseline			1.042	.2836
M6: Indicator for SEQ>19 at Baseline			1.467	.0018
BMAST time dependent			1.046	.2266
SELECT NOT MISSING SEQ, CIGS	514	281		
M2: Continuous SEQ at Baseline				
			1.042	.0040
M2: Continuous SEQ at Baseline			0.900	.4903
CIGS at Baseline				
			1.044	.0027
M3: Continuous SEQ at Baseline			0.788	.1898
CIGS time dependent				
M4: Indicator for SEQ>19 at Baseline			1.550	.0004
M4: Indicator for SEQ>19 at Baseline			1.561	.0003
CIGS at Baseline			0.913	.5433
M6: Indicator for SEQ>19 at Baseline			1.566	.0003
CIGS time dependent			0.822	.2682
SELECT NOT MISSING SEQ, PSYCHWL	491	271		
M1: Continuous SEQ at Baseline			1.040	.0061
M2: Continuous SEQ at Baseline			1.037	.0127
PSYCHWL at Baseline			1.018	.2270
M3: Continuous SEQ at Baseline			1.039	.0073
PSYCHWL time dependent			1.011	.4551
M4: Indicator for SEQ>19 at Baseline			1.462	.0026
M5: Indicator for SEQ>19 at Baseline			1.422	.0066
PSYCHWL at Baseline			1.014	.3413
M6: Indicator for SEQ>19 at Baseline			1.449	.0037
PSYCHWL time dependent			1.007	.6396

SELECT NOT MISSING SEQ, DECLAT	511	280		
M1: Continuous SEQ at Baseline			1.037	.0099
M2: Continuous SEQ at Baseline			1.037	.0099
DECLAT at Baseline			1.001	.9387
M3: Continuous SEQ at Baseline			1.037	.0110
DECLAT time dependent			0.997	.7807
M4: Indicator for SEQ>19 at Baseline			1.484	.0014
M5: Indicator for SEQ>19 at Baseline			1.484	.0014
DECLAT at Baseline			1.000	.9831
M6: Indicator for SEQ>19 at Baseline			1.484	.0014
DECLAT time dependent			0.995	.6530
SELECT NOT MISSING SEQ, ANX	513	280		
M1: Continuous SEQ at Baseline			1.042	.0051
M2: Continuous SEQ at Baseline			1.039	.0100
ANX at Baseline			1.019	.0614
M3: Continuous SEQ at Baseline			1.041	.0068
ANX time dependent			1.005	.6862
M4: Indicator for SEQ>19 at Baseline			1.513	.0008
M5: Indicator for SEQ>19 at Baseline			1.460	.0027
ANX at Baseline			1.016	.1265
M6: Indicator for SEQ>19 at Baseline			1.504	.0010
ANX time dependent			1.005	.6649

Note: SEQ=Sexual Harassment Scale (19-57). DEPR=depressive symptoms. DRINKS=# of drinks consumed per day when drinking, past 30 days. ALCDAYS=number of days alcohol consumed in the past 30 days. INTOX=number of times intoxicated, past 12 months. BMAST=Brief Michigan Alcoholism Screening Test (alcohol misuse). CIGS=use of cigarettes in the past 12 months. PSYCHWL=psychological workload. DECLAT=decision latitude (job control). ANX=symptoms of anxiety.

SUPPLEMENTAL TABLE C.<br>Parameter Estimates for Selected Models<br>Proportional Hazards Multiple Regression Models for First Chronic Disease Diagnosis<br>(asthma, cancer, diabetes, cardiovascular or arthritic disease)<br>(N=488; 269 incident events, 219 right-censored; 23 years followup)					
Models with Single Trend Continuous SEQ					
	Beta	SE	Chi-Square	p-value	HR
Model 1:					
SEQ at Baseline[1]	.03681	.01487	6.1287	.0133	1.038
Current Age >=40	.60245	.24614	5.9910	.0144	1.827
Current Age >=50	.73778	.16156	20.8526	<.0001	2.091
Occupation = 1 (ref=4)[4]	-.13474	.24949	0.2917	.5892	0.874
Occupation = 2 (ref=4)[4]	-.54564	.22727	5.7643	.0164	0.579
Occupation = 3 (ref=4)[4]	-.53700	.24541	4.7879	.0287	0.585
Four groups (DF=3)			10.8468	.0126	
Model 2:					
SEQ at Baseline[1]	.03029	.01520	3.9693	.0463	1.031
DEPR at Baseline[2]	.04766	.01684	8.0082	.0047	1.049
Current Age >=40	.62902	.24662	6.5056	.0108	1.876
Current Age >=50	.76964	.16246	22.4425	<.0001	2.159
Occupation = 1 (ref=4)[4]	-.18521	.25012	0.5483	.4590	0.831
Occupation = 2 (ref=4)[4]	-.55748	.22732	6.0140	.0142	0.573
Occupation = 3 (ref=4)[4]	-.60140	.24624	5.9649	.0146	0.548
Four groups (DF=3)			11.0424	.0115	
Model 3:					
SEQ at Baseline[1]	.03021	.01524	3.9289	.0475	1.031
DEPR at Baseline[2]	.04064	.01714	5.6243	.0177	1.041
DRINKS previous year[3]	.11966	.04821	6.1609	.0131	1.127
Current Age >=40	.66492	.24747	7.2196	.0072	1.944
Current Age >=50	.78482	.16234	23.3708	<.0001	2.192
Occupation = 1 (ref=4)[4]	-.16784	.25024	0.4498	.5024	0.845
Occupation = 2 (ref=4)[4]	-.51357	.22849	5.0521	.0246	0.598
Occupation = 3 (ref=4)[4]	-.53897	.24741	4.7459	.0294	0.583
Four groups (DF=3)			8.9676	.0297	
Models with Binary Indicator SEQ>19					
	Beta	SE	Chi-Square	p-value	HR
Model 1:					
SEQ>19 at Baseline[1]	.36272	.12636	8.2404	.0041	1.437
Current Age >=40	.59601	.24581	5.8792	.0153	1.815
Current Age >=50	.74371	.16125	21.2732	<.0001	2.104
Occupation = 1 (ref=4)[4]	-.16833	.24640	0.4667	.4945	0.845

Occupation = 2 (ref=4)[4]	-.61836	.22260	7.7170	.0055	0.539
Occupation = 3 (ref=4)[4]	-.60693	.24170	6.3054	.0120	0.545
Four groups (DF=3)			13.7016	.0033	
Model 2:					
SEQ>19 at Baseline[1]	.30819	.12808	5.7897	.0161	1.361
DEPR at Baseline[2]	.04607	.01692	7.4143	.0065	1.047
Current Age >=40	.61951	.24611	6.3362	.0118	1.858
Current Age >=50	.77367	.16206	22.7904	<.0001	2.168
Occupation = 1 (ref=4)[4]	-.20813	.24723	0.7087	.3999	0.812
Occupation = 2 (ref=4)[4]	-.61118	.22310	7.5051	.0062	0.543
Occupation = 3 (ref=4)[4]	-.65395	.24234	7.2818	.0070	0.520
Four groups (DF=3)			13.2968	.0040	
Model 3:					
SEQ>19 at Baseline[1]	.30532	.12808	5.6827	.0171	1.357
DEPR at Baseline[2]	.03966	.01713	5.3610	.0206	1.040
DRINKS previous year[3]	.11872	.04811	6.0890	.0136	1.126
Current Age >=40	.65450	.24688	7.0282	.0080	1.924
Current Age >=50	.79015	.16198	23.7946	<.0001	2.204
Occupation = 1 (ref=4)[4]	-.19345	.24731	0.6119	.4341	0.824
Occupation = 2 (ref=4)[4]	-.56781	.22424	6.4119	.0113	0.567
Occupation = 3 (ref=4)[4]	-.59300	.24338	5.9365	.5530	0.553
Four groups (DF=3)			10.9470	.0120	

[1]SEQ = Sexual Harassment Scale (19-57)

[2]DEPR = Depressive Symptoms. Selected items from Center for Epidemiologic Studies Depression Scale (0 – 21)

[3]DRINKS = Drinks per day past 30 days (0-7 categories); value as reported for the previous year or, if missing, last previous reported value.

[4]Occupation: 1=clerical/administrative, 2=faculty, 3=graduate students, 4=service/maintenance.

SUPPLEMENTAL TABLE D. Characteristics of Included and Excluded Samples at Baseline of the Study (1996-1997)

Baseline Variables		Analysis Sample (N=525)				Sample Surveyed at T9 but Excluded (N=377)					Sample Not Surveyed at T9 (N=1,590)				
		n	%			N	n	%			N	n	%		
Age	<=30	130	26.6			377	59	15.6			1539	434	28.2		
	31 – 40	167	34.2				105	27.9				458	29.7		
	41 - 50	128	26.3				113	30.0				289	18.8		
	>50	63	12.9				100	26.5				358	23.3		
		mean	SD	min	max	N	mean	SD	min	max	N	mean	SD	min	max
Age	years	38.3	9.6	22	68	1916	40.8	12.1	20	86	1539	40.3	12.3	20	80
		n	%			N	n	%			N	n	%		
Gender	Female	301	57.3			377	225	59.7			1587	810	51.0		
	Male	224	42.7												
Race	White	340	64.7			377	219	58.1			1587	729	45.9		
	Black	87	16.6				96	25.5				360	22.7		
	Hispanic	25	4.8				25	6.6				142	9.0		
	Asian	64	12.2				34	9.0				313	19.7		
	Other	9	1.7				3	0.8				43	2.7		
Occupation Group	Clerical/ Administrative	86	16.4			377	113	30.0			1590	358	22.5		
	Faculty	205	39.0				138	36.6				422	26.6		

	Graduate Students	192	36.6				91	24.1				592	37.2		
	Service/ Maintenance	42	8.0				35	9.3				218	13.7		
		n	%			N	n	%			N	n	%		
Any Sexual Harassment (SEQ)	= 19 (none)	284	54.1			297	162	54.5			1443	868	60.1		
	> 19 (any)	241	45.9				135	45.5				575	39.9		
		mean	SD	min	max	N	mean	SD	min	max	N	mean	SD	min	max
Sexual Harassment Scale (SEQ)	19 to 57	21.3	3.9	19	45	297	20.9	3.1	19	35	1443	21.0	4.0	19	57

SUPPLEMENTAL TABLE E. Statistical Tests Comparing Subsamples at Baseline
Chi-square test results for categorical variables, independent samples t-test results for continuous variables

Baseline Variables		Analysis Sample (N=525) vs. Sample Surveyed at T9 but Excluded (N=377)			Analysis Sample (N=525) vs. Sample Not Surveyed at T9 (N=1,590)			Analysis Sample (N=525) vs. All Excluded (N=1,967)		
		Chi-Sqre	DF	p-value	Chi-Sqre	DF	p-value	Chi-Sqre	DF	p-value
Age	4 categories	33.2765	3	<.0001	30.9315	3	<.0001	27.8903	3	<.0001
		T-score	DF	p-value	T-Score	DF	p-value	T-Score	DF	p-value
Age	continuous	-6.07	748	<.0001	-3.25	1145	.0012	-4.39	1019	<.0001
		Chi-Sqre	DF	p-value	Chi-Sqre	DF	p-value	Chi-Sqre	DF	p-value
Gender	2 categories	0.4978	1	.4805	6.2674	1	.0123	3.5789	1	.0585
Race	5 categories	14.9360	4	.0048	57.4556	4	<.0001	45.9476	4	<.0001
Occupation Group	4 categories	29.9557	3	<.0001	38.4172	3	<.0001	34.3912	3	<.0001
Any Sexual Harassment (SEQ)	2 categories	0.0155	1	.9009	5.8191	1	.0159	4.3069	1	.0380
		T-Score	DF	p-value	T-Score	DF	p-value	T-Score	DF	p-value
Sexual Harassment Scale (SEQ)	continuous	1.43	727	.1545	1.39	1966	.1653	1.52	2263	.1280
